# The Pro-Coagulant Fibrinogenolytic Serine Protease Isoenzymes Purified from *Daboia russelii russelii* Venom Coagulate the Blood through Factor V Activation: Role of Glycosylation on Enzymatic Activity

**DOI:** 10.1371/journal.pone.0086823

**Published:** 2014-02-10

**Authors:** Ashis K. Mukherjee

**Affiliations:** 1 Department of Molecular Biology and Biotechnology, Tezpur University, Tezpur, Assam, India; 2 School of Biological Sciences, University of Northern Colorado, Greeley, Colorado, United States of America; University Hospital Medical Centre, Germany

## Abstract

Proteases from Russell's viper venom (RVV) induce a variety of toxic effects in victim. Therefore, four new RVV protease isoenzymes of molecular mass 32901.044 Da, 333631.179 Da, 333571.472 Da, and 34594.776 Da, were characterized in this study. The first 10 N-terminal residues of these serine protease isoenzymes showed significant sequence homology with N-terminal sequences of snake venom thrombin-like and factor V-activating serine proteases, which was reconfirmed by peptide mass fingerprinting analysis. These proteases were found to be different from previously reported factor V activators isolated from snake venoms. These proteases showed significantly different fibrinogenolytic, BAEE-esterase and plasma clotting activities but no fibrinolytic, TAME-esterase or amidolytic activity against the chromogenic substrate for trypsin, thrombin, plasmin and factor Xa. Their *Km* and *Vmax* values towards fibrinogen were determined in the range of 6.6 to 10.5 µM and 111.0 to 125.5 units/mg protein, respectively. On the basis of fibrinogen degradation pattern, they may be classified as A/B serine proteases isolated from snake venom. These proteases contain ∼42% to 44% of N-linked carbohydrates by mass whereas partially deglycosylated enzymes showed significantly less catalytic activity as compared to native enzymes. *In vitro* these protease isoenzymes induce blood coagulation through factor V activation, whereas *in vivo* they provoke dose-dependent defibrinogenation and anticoagulant activity in the mouse model. At a dose of 5 mg/kg, none of these protease isoenzymes were found to be lethal in mice or house geckos, suggesting therapeutic application of these anticoagulant peptides for the prevention of thrombosis.

## Introduction

The Russell's viper (*Daboia russelii russelii*), one of the deadliest venomous snakes of the Viperidae family, is widespread in most of the South-East Asian countries [1]. The Russell's viper (RV) is an occupational health hazard for the rice farmers [1], [2]. Snakebite is accountable for a profound toll of human life in the Indian subcontinent, but mournfully effective treatment of snakebite still remains a challenge in the tropical countries [3]. RVs from South-East Asian countries are classified into 5 sub-species; the populations from India, Pakistan and Bangladesh are represented by *Daboia russelii russelii* [4]. It is now well established that differences in venom composition among RVs are associated with their zoogeographic origins, which in turn displays significant variations in the clinical manifestations in RV bite in the South-East Asian countries [1].However, interference in blood coagulation is the most common symptom in RV envenomation, which is independent of geographical origins and subspecies. [1],[2]. Therefore, characterization of RV venom (RVV) components affecting blood coagulation cascade; understanding their molecular mechanisms of action; and assessing the ability of commercial antivenom to neutralize these venom components will be highly useful in hospital management of RV envenomed patients as well as in exploring possible therapeutic applications of such components of venom.

The RVV is enriched in different serine and metallo-proteases, and these components interfere mostly with the haemostatic system of the victim by peptide bond cleavage of one or more specific components of the coagulation cascade [5]–[7]. In addition, venom proteases are reported to induce a variety of toxic effects such as tissue-hemorrhage, myotoxicity, activation of complement system, platelet aggregation and edema–induction in victims or experimental animals [5]–[7].The snake venom serine proteases (SVSPs) belong to the trypsin family S1 of clan SA and represent the largest family of peptidases [8]. Despite sharing a high degree of sequence similarity in their primary structure, relatively minor surface residue changes can result in significant differences in their preference for hydrolyzing a particular macromolecular substrate [7], [8]. Several proteases such RVV -factor X activator (RVV-X), RVV -factor V activator (RVV-V), RVV basic metalloprotease (RVBCMP), and thrombin-like serine protease (Russelobin) have been isolated and characterized from RVV [6], [7], [9], [10]; however, snake venom is reported to contain numerous protease enzymes having distinct biological functions [11]–[13]. Therefore, there are definite prospects of identification and functional characterization of several uncharacterized, new proteases in the venom of RVs from different geographical locations.

The present study is the first report on the purification, biochemical and pharmacological characterization of Factor V activating, pro-coagulant serine protease isoenzymes showing different pharmacological potency from the venom of *D. r. russelii*. Furthermore, molecular characterization revealed that the protease isoenzymes reported in this study are different in several respects from previously reported factor V activating proteases such as RVV-V isolated from *D. r. russelii* [10], and VLFVA isolated from *Vipera labetina* [13]. Moreover, present study is also the first report showing the *in vitro* and *in vivo* pharmacological properties of FV activating serine proteases isolated from RVV.

## Materials and Methods

Pre-cast NuPAGENovex® Bis-Tris Mini Gels, buffers and Mark 12 unstained molecular mass standards were obtained from Life Technologies, Invitrogen Inc, USA. RV (*D. r. russelii*) venom of Pakistan origin was a gift from the Kentucky Reptile Zoo, USA. Protein concentration standard reagents were purchased from BioRad Inc, USA. The deglycosylation mix kit was purchased from New England Biolabs, Inc, USA. The proteomics grade trypsin was obtained from Promega, USA. Lyophilized monovalent antivenom (against *D. r. russelii* venom) was obtained from Vins Bioproducts Limited, India (batch no: 30AS11001; expiry date: 04/2015). Cell culture media was supplied by Invitrogen Inc, USA. All other chemicals used were of analytical grade and were procured from Sigma-Aldrich, USA.

### Purification of coagulant proteases from RVV

Lyophilized *D. r. russelii* venom (200 mg dry weight) dissolved in 25 mM HEPES buffer containing 100 mM NaCl and 5 mM CaCl_2_ (pH 6.8) was fractionated through size-exclusion column (BioGel P-100) as described by us [7]. The tubes were screened for coagulant as well as for protease activities. The gel-filtration tubes 58–62 showing strong plasma clotting, and displaying protease and BAEE-esterase activities were pooled, desalted by dialyzing (3.5 kDa cut-off membrane, Spectrum Laboratories, INC) and was then lyophilized. The freeze-dried sample was dissolved in 0.5 ml of buffer A (20 mM Tris-HCl, pH 8.0) and was then subjected to second chromatographic separation by using a FPLC-Mono Q 5/50 GL anion exchange chromatography (AKTA Purifier Fast Protein Liquid Chromatography System, GE Healthcare). After eluting the non-bound proteins with 3 column volume of equilibration buffer, the bound proteins were fractionated with a linear gradient from 0 to 350 mM NaCl in 20 mM Tris-HCl, pH 8.0 (buffer B) at a flow rate of 45 ml/ h for 80 min. Elution of proteins was monitored at 280 nm, and the fraction volume was 0.75 ml. The fractions displaying coagulant activity were subjected to further study.

The protein peaks were desalted, lyophilized and were then re-dissolved in a minimum volume of buffer A, and the protein content was determined with the help of the Bio-Rad protein assay kit (BIO-RAD, USA) using bovine serum gamma globulin as a standard. The homogeneity and molecular mass of each protein peak was determined by 12.5% SDS-PAGE of reduced and non-reduced proteins as well as by MALDI-TOF-MS as described by Mukherjee and Mackessy [7].

### N-terminal sequencing and peptide mass fingerprinting

About 5 µg of FPLC purified protein was blotted into PVDF membrane, and then N-terminal sequencing was done by Edman degradation on a Protein Sequencer (ABI). The online BLASTP (Basic Local Alignment Search Tool) program of the National Center for Biotechnology Information (www.ncbi.nlm.nih.gov) was used to search the protein homology against the snake venom proteins (taxid 8570) deposited in the non-redundant protein sequences (nr) databases. Multiple alignments of homologous sequences from snake venoms were performed by using COBALT (Constraint-based Multiple Alignment; NCBI).

The purified protein was in-gel alkylated, reduced and was then tryptic digested for 16 h at 37°C [7]. The MS/MS spectra of tryptic digested peptides were searched against the NCBI data base of non-redundant protein sequence (NCBI nr) using the Mascot database search engine (version 2.3) as described by us [7],[14]. The *de novo* sequences of the peptides obtained from Mascot protein identification were subjected to a BLAST search in NCBInr against a snake venom protein database (snakes, taxid: 8570) using the BLASTP algorithm (http://blast.ncbi.nlm.nih.gov/Blast.cgi) [7].

### Assay of amidolytic, esterase, protease activity and substrate specificity

The amidolytic activity of gel-filtration fraction as well as of purified proteases against selected chromogenic substrates (final concentration 0.2 mM) was assayed by the method as described by Mukherjee and Mackessy [7]. The unit of amidolytic activity has been defined as µmoles of 4-nitroaniline released per minute by the protease under the assay conditions [7]. The esterolytic activity was assayed by using *N_α_*-*p*-tosyl-L-arginine methyl ester hydrochloride (TAME) or *N*_α_-benzoyl-L-arginine ethyl ester hydrochloride (BAEE) as substrate by following our previously described procedures [7]. One unit of TAME or BAEE-esterase activity has been defined as an increase in absorbance of 0.01 at 244 or 254 nm, respectively during the first 5 min of the reaction at 37°C [7].

The proteolytic activity of purified protease isoenzymes against the protein substrates (1%, w/v) casein, bovine serum albumin, bovine serum gamma globulin, human plasma fibrinogen (fraction I) and fibrin was determined by a modification of the method described by us [14].One unit (U) of protease activity has been defined as 1.0 µg of tyrosine equivalent liberated per min per ml of enzyme. The fibrinogen degradation pattern of purified proteases (0.25 to 2 µM) for 5 h at 37°C was also determined by SDS-PAGE analysis [7],[14]. The peptide bond specificity of the protease isoenzymes against the oxidized B-chain of bovine insulin was determined by RP-HPLC analysis of insulin B-chain degradation products as described by Weldon and Mackessy [15].

### Assay of plasma clotting, factor V activation and *in vitro* pharmacological properties

Dose-dependent anticoagulant or hemolytic activity of purified proteases (12.5 to 200 nM) was analysed, respectively, against platelet poor plasma and 5% (v/v) washed erythrocytes isolated from goat blood following our previously described procedures [7]. The prothrombin activating property (FXa-like activity) of the purified proteases was assayed by determining the amount of thrombin formed from prothrombin using chromogenic substrate (N-p-tosyl-Gly-Pro-Arg p-nitroanilide acetate salt) for thrombin. Briefly, after incubating the purified protease (250 nM) with prothrombin (1.4 µM) for 30 min at 37°C, the above chromogenic substrate (0.2 mM) was added and the rate of thrombin formation was monitored by measuring the initial rate of p-nitroaniline liberation at 405 nm. As a positive control, prothrombin was incubated with Factor Xa (0.15 nM) under identical conditions.

For studying the FV activating property of purified proteases, the collagen (5 µg)-induced factor V release from platelets was done as described by Moncovik and Tracy [16] and the concentration of factor V was determined as described by Gerads et al [17].The active factor V (FVa) was prepared by incubating FV with FXa at 23°C for 15 min [16]. For assaying the FV activation by RVV proteases, platelet factor V (16 nM) was incubated with purified protease (50 nM) for 30 min at 37°C in 20 mM Tris-HCl, 150 mM NaCl, pH 7.4. Thereafter, FXa (0.15 nM), 50 µM phospholipids vesicles (9∶1 PC: PS) and 2.0 mM CaCl_2_ were added, mixed well and incubated for 5 min at 23°C. The reaction was initiated by adding 1.4 µM human prothrombin (pre-incubated for 5 min at 37°C) and 0.2 mM chromogenic substrate (N-p-tosyl-Gly-Pro-Arg p-nitroanilide acetate salt) for thrombin. The increase in absorbance due to thrombin catalyzed release of pNA was continuously recorded at 405 nM up to 5 min in a microplate reader. In a separate set of experiments, RVV protease-activated FV was replaced either by factor V or FXa-activated factor V (FVa) and prothrombin activation assay was done under identical experimental conditions.

The fibrinogen clotting ability of the purified protease isoenzymes was also determined by using a BBL-Fibrinosystem, as described earlier [7]. Furthermore, in order to detect the release of fibrinopeptides A and B from fibrinogen after incubation with RVV protease, the fibrinogen degradation products were separated by RP-HPLC followed by MS analysis of peaks [7].To study the effect of pre-incubation of fibrinogen with purified RVV proteases on thrombin clotting activity, 250 nM of protease was incubated with 40 µl of fibrinogen solution (2.5 mg/ml in 20 mM K-phosphate buffer, pH 7.4) for 30 to 60 min at 37°C. Thereafter, 3 µl of thrombin (10 NIH U/ ml) was added to the reaction mixture and the time of clot formation was recorded. A control was run in parallel where instead of purified protease buffer was added to the fibrinogen solution and thrombin clotting time was recorded.

Dose-dependent *in vitro* cytotoxicity against Colo-205 (human colorectal adenocarcinoma) and MCF-7 (human breast adenocarcinoma) was assayed by adding purified protease (250 nM) to the culture medium containing 1×10^5^ cells/ml [7]. Cytotoxicity (percent cell death) was assayed by an MTT-based method. Protease-induced cytotoxicity, if any, was expressed as percent cell death as determined by comparison with values obtained from a standard curve of control cells [7].

### Determination of biochemical properties, carbohydrate content and role of glycosylation on enzyme activity

The Bio-Rad protein assay kit (BIO-RAD, USA) was used for assaying the protein content against bovine serum gamma globulin as a standard. Optimum conditions for protease activity were determined by incubating 100 nmol of enzyme at different pH (7–9.5) and temperature ranges (30–55°C), which was followed by a measuring of the fibrinogenolytic activity of each enzyme as described above. The effect of the five cycles freeze-thawing on protease activity was determined by our previously elucidated procedure [7]. Kinetics parameters were calculated by incubating enzyme (500 nM) with different concentrations of fibrinogen (1.0 to 5 µM) for 3 h at 37°C, and the *K_m_* and *V_max_* values were calculated from a double reciprocal (Lineweaver-Burke) plot. Similarly, the kinetics parameters for BAEE-esterase activity were determined by incubating 50 nM of enzyme with graded concentrations (0.04 to 0.2 mg/ml) of BAEE and then the *K_m_* and *V_max_* values were calculated as stated above.

The phenol-sulfuric acid colorimetric method was used to determine the total neutral sugar content of the proteases [18]. The extent of N-linked or O-linked oligosaccharides as well as sialic acid content was determined by incubating 2 µg of denatured enzyme with PNGase F (N-glycosidase), O-glycosidase and neuraminidase, respectively for 4 h at 37°C following the instructions of manufacturer. The reaction products were visualized by 12.5% NuPage SDS-PAGE under reducing conditions [7]. Partial deglycosylation without denaturing the enzymes (native deglycosylation) was achieved by eliminating the denaturation step and treating the proteases with 2000 units of PNGase F for 24 at 37°C. A control was also run in parallel where the protease was treated with buffer instead of PNGase F. Differences in biochemical properties (fibrinogenolytic and BAEE-esterase activities, optimum temperature and pH, and thermostability) between the native (glycosylated) and partially deglycosylated enzymes were then compared.

### Effect of chemical inhibitors and antivenom on fibrinogenolytic, esterase and plasma clotting activity of proteases

Purified protease (500 nM) was pre-incubated with one of the following inhibitors (final concentration) for 30 min at 37°C before assaying the inhibition of its fibrinogenolytic, esterase and pro-coagulant activities: benzamidine-HCl (5 mM), aprotinin (100 µM), diNa-EDTA (5 mM), α_2_-macroglobulin (100 µg), TPCK (100 µM), TLCK (100 µM), 4-(2-aminoethyl) benzene sulfonyl fluoride hydrochloride (5 mM) and graded concentrations of commercial monovalent antivenom against RVV. After incubation, protease was assayed for its catalytic (fibrinogenolytic and BAEE-esterase) and coagulant activities in the corresponding assay system. The activity of the respective protease in the absence of an inhibitor was considered 100% activity and other values were compared with that.

### Determination of *in vivo* toxicity

All the animal experimental protocols were approved by the University of Northern Colorado IACUC (protocol/permit 9401). To determine the *in vivo* toxicity, purified protease isoenzyme (in 0.2 ml of PBS, pH 7.4) was injected i.p (1.0 mg/kg to 5.0 mg/kg body weights) into the laboratory inbred, non-Swiss NSA strain albino mice (n = 3) weighing between 18–20 g. Similarly, house geckos (*Hemidactylus frenatus*) weighing between 1.5–3.5 g was also injected with the same dose in a total volume of 75 µl. The control group of mice or house geckos received only 0.2 ml or 75 µl of PBS, pH 7.4 (placebo). The treated-animals were observed at regular intervals up to 72 h post-injection for death or any physical or behavioral changes [7].

The *in vivo* defibrinogenating activity as well as *in vitro* blood clotting time of protease-treated mice and control group of mice were determined 6 h after i.p injection of purified protease isoenzyme at two doses (2.0 and 4.0 mg/kg). The fibrinogen content of plasma was determined by using a Ca-thrombin reagent as described by Burmester et al [19]. After the end of experiment, the mice were euthanized with an over- dose of Na-pentobarbital as per the recommendations of the American Veterinary Medical Association (AVMA) Panel on Euthanasia, 2007.

### Statistical analysis

Student's t test using the software SigmaPlot 11.0 for Windows (version 7.0) was used to determine the significance of difference between two test values. The value of p≤0.05 was considered significant.

## Results

### Purification of coagulant protease isoenzymes from RVV

**Figure 1:**
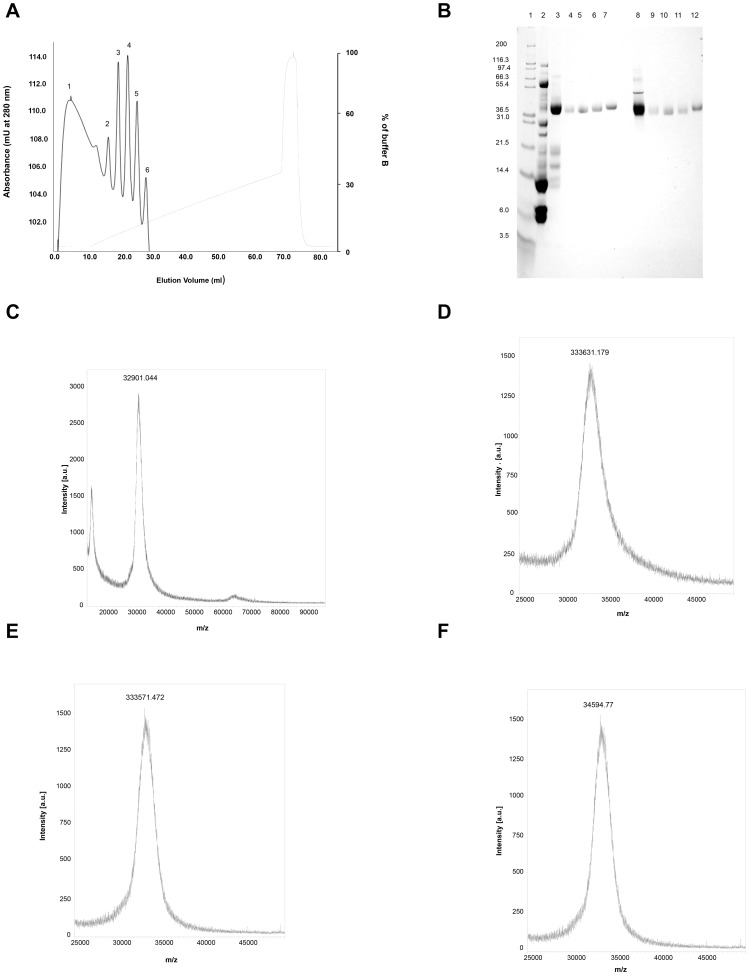
A. Purification of protease isoenzymes from Russell's viper venom. Separation of RVV gel-filtration (GF 58–62) fractions through FPLC Mono Q 5/50 GL column. After eluting the non specifically bound proteins with buffer A (20 mM Tris-HCl, pH 8.0), bound proteins were eluted with 350 mM NaCl in buffer A. The coagulant protease isoenzymes were eluted in peaks 3 (RV-FVP_α_), 4 (RV-FVP_β_), 5 (RV-FVP_γ_), and 6 (RV-FVP_δ_). B. Assessment of purity and molecular masses of protease isoenzymes. The purity and molecular mass were determined by SDS-PAGE of proteins under reduced (lanes 2–7) and non-reduced (lanes 8–12) conditions. Lane 1, protein molecular markers; lane 2, crude RVV (20 µg); lanes 3 and 8, gel-filtration fraction (8 µg); lanes 4 and 9, RV-FVP_α_ (2.0 µg); lanes 5 and 10, RV-FVP_β_ (2.0 µg); lanes 6 and 11, RV-FVP_γ_ (2.0 µg); lanes 7 and 12, RV-FVP_δ_ (2.0 µg). C–F. Mass spectroscopic determination of molecular mass of purified protease isoenzymes. The mass of purified protease isoenzymes was determined by MALDI-TOF-MS analysis of 1.0 µg of protein as described in the text; (C) RV-FVP_α_, (D) RV-FVP_β_, (E) RV-FVP_γ_, (F) RV-FVP_δ_.

Fractionation of proteins of gel-filtration tubes 58–62 (see Fig. 1a of reference 7) on a FPLC-Mono Q 5/50 GL column resulted in their separation into 6 peaks. The FPLC peaks 3 to 6 showed appreciable BAEE-esterase and plasma clotting activity (**Fig 1A**) and the proteins of these peaks were therefore subjected to further study. All these four peaks displayed a single but slightly diffused band in SDS-PAGE under both reduced and non-reduced conditions, suggesting that all these proteins are monomeric glycoproteins (**Fig. 1B**). By SDS-PAGE analysis, these proteins showed molecular masses in the range of 38 to 40 kDa (**Fig. 1B**). By MALDI-TOF-MS, the molecular masses of FPLC peaks 3, 4 5, and 6 were determined as 32901.044 Da, 333631.179 Da, 333571.472 Da, and 34594.776 Da, respectively (**Figs 1C**–**F**). These proteins are named RV-FVP_α_, RV-FVP_β_, RV-FVP_γ_ and RV-FVP_g_, respectively. A summary of the purification of these protease isoenzymes is shown in **Table S1 in File S1**.

### N-terminal sequencing and PMF analysis

The N-terminal sequences (10 amino acid residues) of the purified proteases were found to be 100% identical with each-other. Furthermore, they showed 100% sequence homology to N-terminal sequences of previously reported thrombin-like enzymes/ serine proteases and alpha-fibrinogenases from snake venom (**Table 1**). In addition, N-terminal sequences of four purified proteins also showed 100% sequence homology with *Vipera* (*Daboia*) *russelii* proteinase RVV-V homolog 1 (Accession no. P86530.1, **Table 1**) and 90% identity with the previously described serine protease Russelobin isolated from the same venom [7]. Furthermore, except Russelobin, the molecular mass of all these proteases by SDS-PAGE analysis was determined in the range of 30 to 40 kDa (**Table 1**).

**Table 1:** Multiple sequence alignment of N-terminal sequence of RVV protease isoenzymes with other known serine proteases isolated from snake venom.

Accession/Reference	Description	Snake species	N-terminal sequence 1 10	Identity (%)	Molecular mass in kDa (SDS-PAGE)
Present study	RV-FVP_α_	*D. r. russelii*	VIGGDECNIN	-	38
-do-	RV-FVP_β_	*D. r. russelii*	VIGGDECNIN	100	39
-do-	RV-FVP_γ_	*D. r. russelii*	VIGGDEXNIN	100	39
-do-	RV-FVP_δ_	*D. r. russelii*	VIGGDECNIN	100	40
P0DKX2.1	Thrombin-like enzyme Cdc SI	*Crotalus durissus cumanensis*	VIGGDECNIN	100	28
Q9PRW2.1	Alpha-fibrinogenase A3	*Crotalus atrox*	VIGGDECNIN	100	35
P86530.1	*Vipera russelli* proteinase RVV-V homolog 1	*D. r. russelli*	VIGGDECNIN	100	29
P81882.1	Thrombin-like enzyme TL-BJ 1	*Bothrops jararaca*	VIGGDECNIN	100	30
P0C590.1	Thrombin-like enzyme calobin-2	*Gloydius ussuriensis*	VIGGDECNIN	100	41
P20005.1	Thrombin-like enzyme okinaxobin-1	*Ovophis okinavensis*	VIGGDECNIN	100	37.5
Q9PRW4.1	Serine protease α-fibrinogenase A1	*Crotalus atrox*	VIGGDECNIN	100	33
P0DJG6.1	Snake venom serine protease	*C. viridis viridis*	VIGGDECNIN	100	
Mukherjee and Mackessy [7]	Russelobin	*D. r. russelii*	VVGGDECNIN	90%	51.3
Siigur et al.[13]	Factor V activator (VLFVA)	*Vipera lebetina*	VVGGDECDIN	90%	∼30

NR: not reported.

By PMF analysis, no putative conserved domain was recognized and no significant hit could be generated for any of the purified protease isoenzyme. Nevertheless, in addition to some distinct peaks, MALDI-TOF-MS analysis of tryptic digested RV-FVP_α_, RV-FVP_β_, RV-FVP_γ_ and RV-FVP_δ_ showed predominance of three peptides at m/z 804.6065, 825.3817 and 841.3000. The BLASTP analysis of some of the tryptic peptide sequences of these protease isoenzymes demonstrated similarities to thrombin-like enzymes, venom prothrombin activators, dipeptidylpeptidase, bradykinin-potentiating peptide and serine proteases from snake venom (**Table S2 in File S1**).

### Esterase, protease and amidolytic activity

All the four protease isoenzymes showed BAEE-esterase activity but none of them displayed TAME-esterase activity. The protease RV-FVP_α_ showed highest specific activity in hydrolyzing the BAEE as compared to the remaining three protease isoenzymes which showed comparable BAEE-esterase activity (**Table S1 in File S1**). The *Km* value of RV-FVP_α_, RV-FVP_β_, RV-FVP_γ_ and RV-FVP_δ_ towards BAEE was determined as 0.143 mg/ml, 0.156 mg/ml, 0.159 mg/ml, and 0.159 mg/ml, respectively (data not shown).

The specific activity of these protease isoenzymes against various protein substrates is shown in **Table 2**. Hydrolysis of casein or bovine serum albumin was shown only by RV-FVP_α_ but none of the protease isoenzymes showed hydrolysis of azocasein or bovine serum globulin (**Table 2**). These protease isoenzymes demonstrated hydrolysis of human plasma fibrinogen after 3 h of incubation at 37°C albeit to a significantly different extent (p<0.05). Among these proteases, the fibrinogenolytic activity of RV-FVP_α_ was found to be the highest whereas RV-FVP_δ_ showed the least fibrinogenolytic activity under identical experimental conditions (**Table 2**).

**Table 2:** Activity of protease isoenzymes against various protein and chromogenic substrates.

Protein substrate	Specific activity (U/mg protein)				
	GF fraction	RV-FVP_α_	RV-FVP_β_	RV-FVP_γ_	RV-FVP_δ_
Casein	1.9±0.3	3.4±0.3	0	0	0
Azocasein	0	0	0	0	0
Bovine serum albumin	2.9±0.5	5.1±0.4	0	0	0
Bovine serum globulin	0	0	0	0	0
Bovine fibrinogen	0	2.1±0.4^a^	0.8±0.1^b^	0.7±0.1^b^	0.4±0.05^c^
	1.8±0.2# ^, a^	15.6±1.1^b^	9.8±1.2^c^	7.9±0.9^d^	4.5±0.5^e^
Bovine fibrinogen†	ND#	10.1±0.8^a^	5.8±1.3^b^	3.9±0.6^b^	1.1±0.2^c^
Fibrin	0#	0	0	0	0
**Chromogenic substrates**					
Nα-Benzoyl-L-arginine pNA.HCl^1^	1.3±0.1	1.3±0.2	0	0	0
N-Bz-Phe-Val-Arg-pNA.HCl^2^	1.4±0.3	0.2±1.3	0.2±0.02	0.14±.02	0
N-α-tosyl-Gly-pro-Arg-pNA^3^	0.44±0.01	0.3±0.01	0.4±0.01	0.5±0.04	0
D-Val-Leu-Lys-pNA^4^	0.5±0.02	0.23±0.02	0	0	0
N-Bz-Ile-Glu-Gly-Arg p-nitroanilide acetate^5^	0.05±0.01	0.5±0.03	0.04±0.01	0.08±.02	0.1±0.03
**Kinetic for fibrinogenolytic activity**					
*Km* (µM)	ND	6.6	9.52	10.0	10.5
*Vmax* (µg L-tyrosine/min/mg protein)	ND	125.0	122.0	121.0	111.0
*Kcat* (min^-1^)	ND	62.5	20.0	20.2	18.5
**Carbohydrate content**					
(a) Neutral (µg glucose/ mg protein)	ND	33.3±2.1^a^	207±4.3^b^	41±1.9^c^	238±3.6^d^
(b) N-linked oligosaccharides (% by mass)	ND	∼42-43	∼42–43	∼43–43	∼43–44

† Specific activity of partially deglycosylasted protease isoenzymes; ND: not determined.

# Incubated for 180 min at 37°C.

Substrate(s) for ^1^trypsin, ^2,3^thrombin, ^4^plasmin, ^5^FXa.

Incubation was carried out with 1% (w/v) protein substrate at pH 8.0, 37°C for 90 min or ^#^180 min. For amidolytic activity assay, protease was incubated with 0.2 mM chromogenic substrate for 10 min at 37°C. Values are mean±SD of triplicate determinations. Values in the same row with different subscripts (a–e) are significantly different (P<0.05).

**Figure 2:**
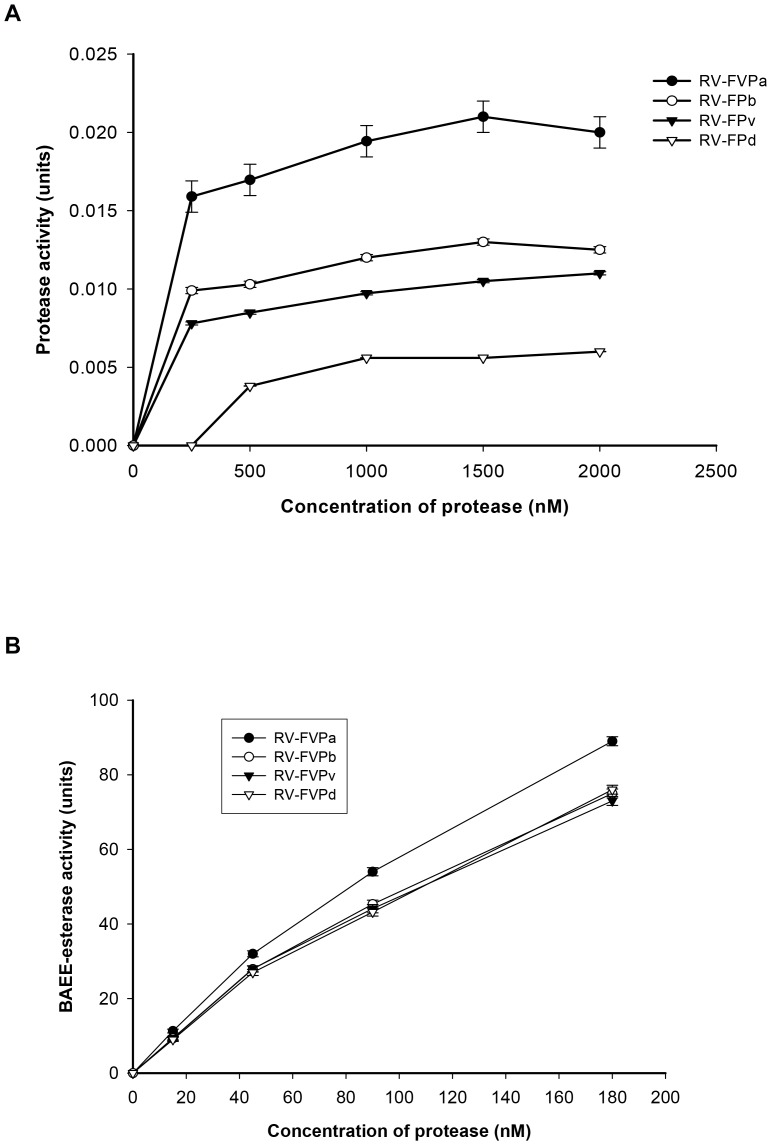
Comparison of dose-dependent fibrinogenolytic and BAEE-esterase activities of protease isoenzymes purified from RVV. Fig.2 (A) shows fibrinogenolytic activity and Fig 2(B) depcits BAEE-esterase activity of purified protease isoenzymes. The values are mean ± S.D of the three experiments.

**Figure 3:**
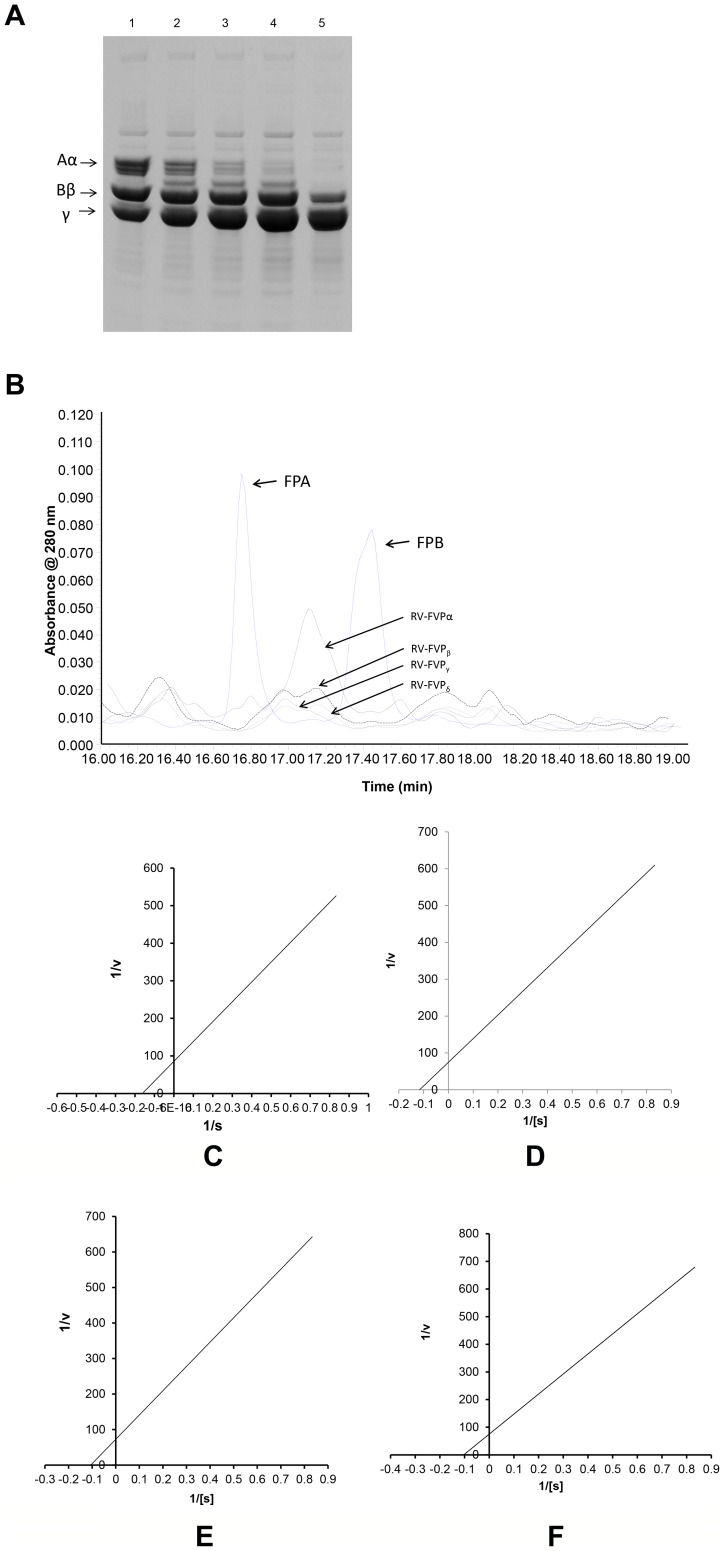
A. Assessment of dose-dependent fibrinogenolytic activity of RV-FVP_α_ by SDS-PAGE analysis. The dose-dependent fibrinogenolytic activity of RV-FVP_α_ was analyzed by 12.5% SDS-PAGE under reduced conditions. Lane 1, control fibrinogen; lanes 2 to 5, fibrinogen was incubated with RV-FVP_α_ at a concentration of 250 nM, 500 nM, 1000 nM, and 2000 nM, respectively for 5 h at 37°C. B. RP-HPLC analysis of RVV protease isoenzymes (500 nM) catalyzed human fibrinogen degradation products. Incubation of fibrinogen with protease isoenzymes was carried out for 5 h at 37°C. Elution profile of FPs A and B (1 µg each) under identical RP-HPLC conditions is also shown. C–F. Determination of kinetic parameters of fibrinogenolytic activity of protease isoenzymes purified from RVV. Double-reciprocal plot was drawn to determine the Kinetic parameters (*Km and Vmax*) of fibrinogenolytic activity of (C) RV-FVPα, (D) RV-FVP_β_, (E) RV-FVP_γ_, and (F), RV-FVP_δ_. Incubation of protease (500 nM) with different concentrations fibrinogen (1 to 5 µM) was carried out for 3 h at 37°C.

All the proteases showed dose-dependent fibrinogenolytic and BAEE-esterase activities (**Figs 2A**–**B**).The dose-dependent fibrinogenolytic activity of RV-FVP_α_ by SDS-PAGE analysis demonstrated that at a dose of 250 nM, only the Aα-band of fibrinogen was degraded whereas the Bβ and γ-chains of fibrinogen remained unaffected (**Fig. 3A**). With a further increase in concentration of RV-FVP_α_ to 2 µM, the Bβ-chain of fibrinogen was partially degraded; nevertheless, the γ-chain of fibrinogen remained intact (**Fig 3A**). The other three protease isoenzymes also displayed the same result, albeit at a much higher protein concentration as compared to RV-FVP_α_ (data not shown). By RP-HPLC analysis, none of the protease isoenzymes was found to release fibrinopeptide A or fibrinopeptide B from fibrinogen (**Fig. 3B**).Further, none of the protease isoenzymes degraded the fibrin (**Table 2**) or showed cleavage of insulin B-chain.

The amidolytic activities of gel-filtration fraction and purified protease isoenzymes from RVV against various chromogenic substrates are shown in **Table 2**. All the purified protease isoenzymes including the gel-filtration fraction showed insignificant amidolytic activity against the chromogenic substrates for trypsin, thrombin, plasmin and FXa (**Table 2**).

### Biochemical properties and role of glycosylation

All the purified protease isoenzymes showed optimum activity (fibrinogenolytic as well as BAEE-esterase) at pH 8.0 and 37°C. They were fully stable against freeze-thawing as after 5 cycles of freeze-thawing the enzymes retained 96±1 % (mean ± SD, n = 3) of their original protease activity. Furthermore, all these protease isoenzymes were found to be thermostable because heating them at 70°C for 30 min resulted in loss of 8±1.0 % (mean ± SD, n = 3) of original protease activity (data not shown). All the protease isoenzymes demonstrated classic Michaelis-Menton behavior at a fibrinogen concentration of 1 to 5 µM. The kinetics parameters (*Km*, *Vmax* and *Kcat*) for fibrinogenolytic activity (1.0 to 5.0 µM fibrinogen concentrations) of these proteases (500 nM) as determined by LB plot (**Figs. 3C-F**) are displayed in **Table 2**.

**Figure 4:**
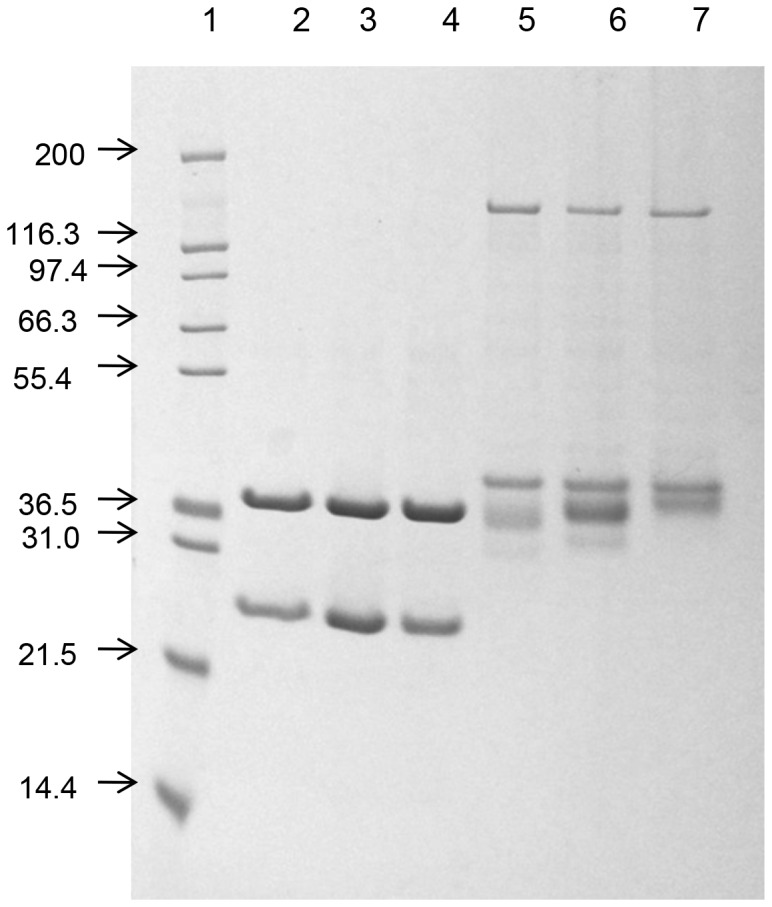
SDS-PAGE analysis of deglycosylated protease isoenzymes. Protease isoenzymes were treated with glycosidase enzymes under reduced conditions for 4°C and were then analyzed by 12.5 % SDS-PAGE. Lane 1, protein molecular markers; lanes 2–4, PNGase F-treated RV-FVPα, RV-FVP_γ_ and RV-FVP_δ_ enzymes, respectively; lanes 5–7, neuraminidase and O-glycosidase-treated RV-FVPα, RV-FVP_γ_ and RV-FVP_δ_ enzymes, respectively.

The neutral carbohydrate content of the proteases is shown in **Table 2**. The RV-FVP_δ_ protease was found to contain the highest amount of neutral carbohydrate whereas RV-FVP_α_ protease was characterized by possessing the least amount of neutral carbohydrate (**Table 2**).Treatment of enzymes with N-glycosidase (PNGase F) under denaturing conditions for the removal of N-liked sugars resulted in partial deglycosylation of enzymes corresponding to protein bands of ∼37 kDa and ∼23 kDa (**Fig.4**). This indicates that the N-glycosylated oligosaccharides constituted about 42% to 44% of the total mass of these proteases. Treatment with neuraminidase or *O*-glycosidase did not result in a significant change in the SDS-PAGE migration pattern of these proteases (**Fig. 4**). The partial deglycosylation of proteases (under denaturing conditions) did not result in changes in their biochemical properties such as temperature and pH optima, and effect of freeze-thawing on catalytic activity (data not shown). However, the fibrinogenolytic activity of the deglycosylated protease isoenzymes significantly dropped (p<0.01) as compared to the same activity displayed by the native, glycosylated enzymes (**Table 2**).

### Plasma coagulant activity and factor V activating property

**Figure 5:**
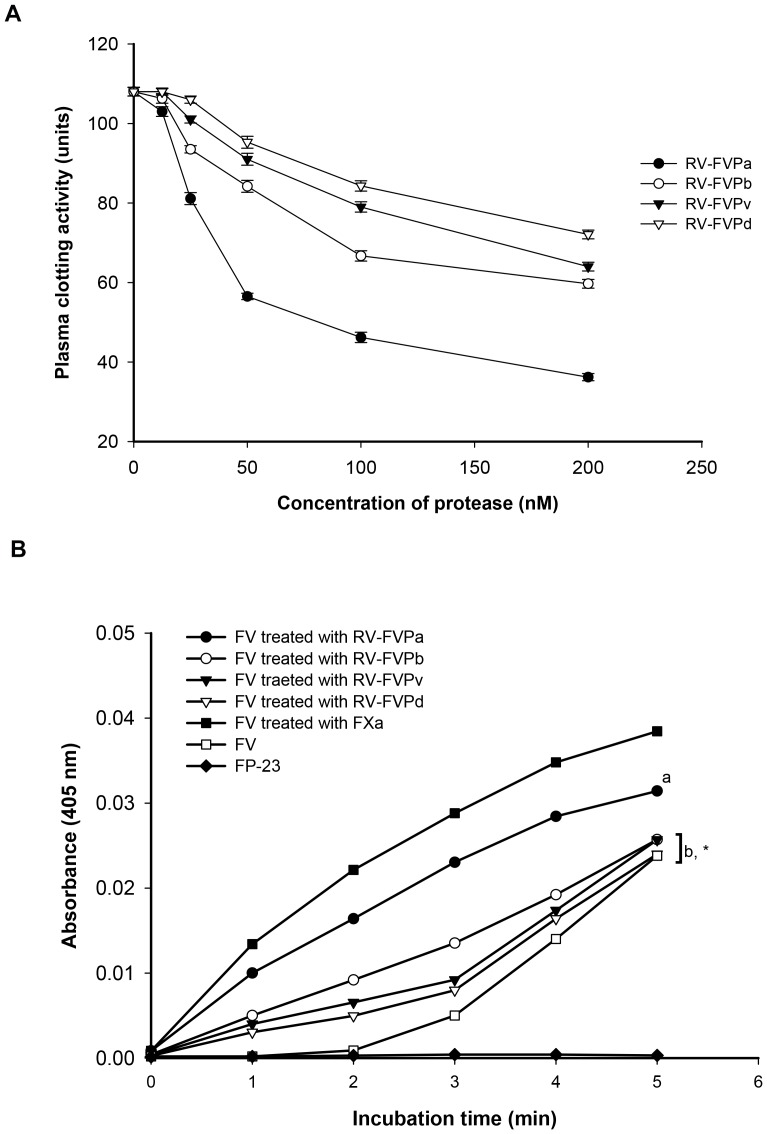
A. The concentration-dependent platelet poor plasma clotting activity of purified protease isoenzymes. The experiment was done as described in the text. The values are mean ± S.D of the triplicate determinations. B. Platelet FV activating property of RVV protease isoenzymes. The experiments were done as described in the text. Values are mean of triplicate determinations. Significance of difference with respect to prothrombin activation by FXa-activated FV (^a^p<0.01; ^b^p<0.001) and RV-FVP_α_-activated FV (*p<0.001). The RV-FVP_α_ or other protease isoenzymes alone did not show prothrombin activating property.

All the protease isoenzymes demonstrated dose-dependent decrease in the Ca-clotting time of platelet poor plasma; however, to a significantly different extent (**Fig 5A**). Among the purified proteas isoenzymess, RV-FVP_α_ showed highest coagulant activity whereas RV-FVP_δ_ demonstrated lowest coagulant activity under identical experimental conditions (**Fig. 5A**). But none of the protease isoenzymes demonstrated fibrinogen clotting activity. The effect of pre-incubation of fibrinogen with RV-FVP_α_ 60 min prior to the addition of thrombin resulted in significant prolongation (p<0.001) of thrombin clotting time of fibrinogen from 39±1.3 sec (control value, mean ± S.D., n = 3) to 360±6 sec (treated value, mean ± S.D., n = 3). Incubation of fibrinogen with other purified protease isoenzymes before addition of thrombin also resulted in significant increase in the thrombin clotting time of protease-treated fibrinogen as compared to control fibrinogen (data not shown).

None of the proteases at a dose of 250 nM exhibited prothrombin activation property. **Fig. 5B** shows a comparison of the prothrombin activating property of factor V treated with protease isoenzyme or FXa. The treatment of FV with RVV -protease isoenzymes/FXa resulted in formation of activated factor V (FVa) which in turn activated FXa to produce thrombin from prothrombin in a quantity higher the quantity of thrombin produced by FV alone under identical experimental conditions (**Fig. 5B**). However, thrombin generation was gradually stimulated after 2 min of incubation of prothrombin with FV along with other components of prothrombinase complex (**Fig. 5B**).

### Effect of inhibitors on fibrinogenolytic, BAEE-esterase and coagulant activities

The effects of inhibitors and monovalent antivenom on the protease, esterase and coagulant activities of RV-FVP_α_ are displayed in **Table 3**. Among the inhibitors tested, serine protease inhibitors benzamidine, AEBSF, and also the disulphide bonds reducing agent DTT significantly inhibited the protease, the BAEE-esterase and the coagulant activities of RV-FVP_α_ (**Table 3**) and also other protease isoenzymes in a parallel manner (data not shown). The monovalent antivenom showed dose-dependent neutralization of the protease; esterase or plasma clotting activity of RV-FVP_α_ (**Table 3**) as well as other protease isoenzymes (data not shown).

**Table 3:** Effect of inhibitors on fibrinogenolytic, BAEE-esterase and plasma clotting activities of RV-FVP_α_.

Inhibitor (final concentration)	Percent activity		
	Fibrinogenolytic	BAEE-esterase	Coagulation
Control	100	100	100
Benzamidine-HCL (5 mM)	13.4±1.1*	10.3±0.6*	17.3±2.1*
AEBSF (5 mM)	9.3±0.6*	7.4±1.2*	12.6±1.1*
Aprotinin (0.1 mM)	103±3.1	98.4±4.1	96.4± 3.2
S. trypsin inhibitor-I (1∶20)	107.3±6.3	100.4±1.7	97.8±2.1
DTT (5 mM)	86.5±2.9*	81.2±2.2*	76.4±2.9*
TPCK (0.1 mM)	100±2.1	106±3.1	99±2.3
TLCK (0.1 mM)	102±4.3	104±2.6	98±1.3
EDTA (5 mM)	97.3±1.7	101±2.1	107±4.3
α2 –macroglobulin (1∶20)	103.5±1.2	99±0.8	106±3.1
Monovalent antivenom (antigen: antivenom)			
1∶1	29.1±1.2*	33.3±2.1*	30.1±0.8*
1∶10	18.4±2.4*	22.4±2.3*	22.3.4±3.3*
1∶30	5.5±0.9*	9.2±1.0*	7.5±1.6*

Values are mean ± SD of triplicate determinations. Significance of difference with respect to control *p<0.01.

### Pharmacological properties, *in vivo* toxicity and defibrinogenating activity

At a dose of 500 nM, except for RV-FVP_α_ no other protease isoenzyme displayed cytotoxicity towards MCF-7 or Colo-205 cells; RV-FVP_α_ at a dose of 250 nM inhibited 31.6±2.1 and 6.3±0.8 % (mean± SD, n = 3) growth of MCF-7 and Colo-205 cells, respectively after 72 h of incubation.

**Figure 6:**
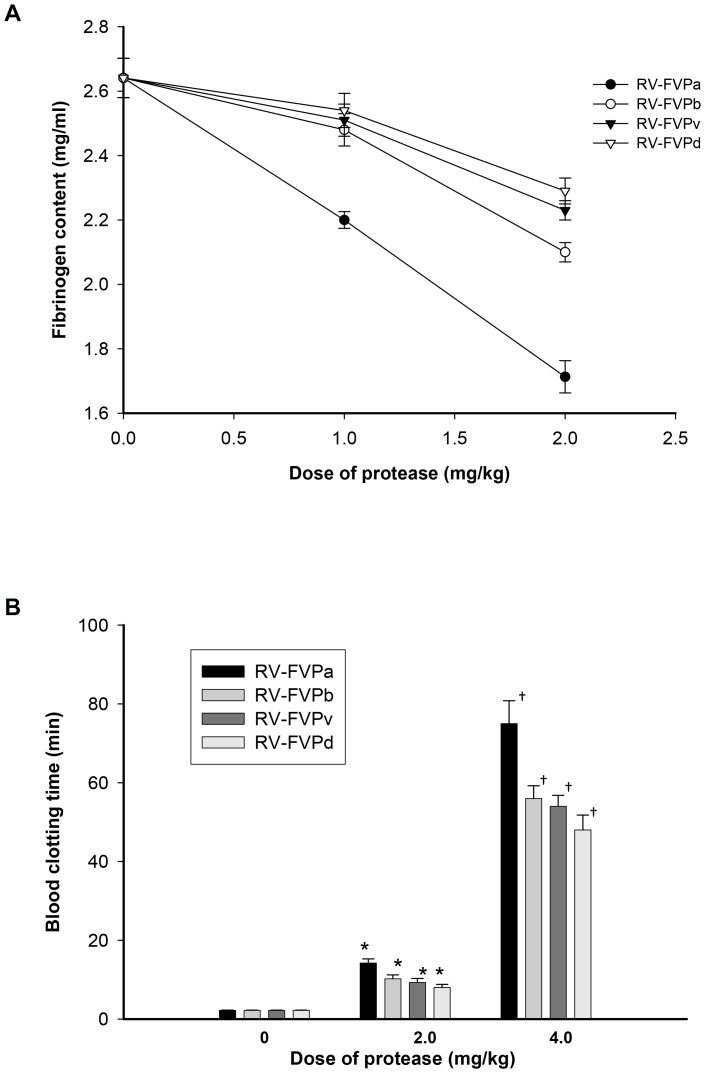
Dose-dependent *in vivo* defibrinogenating activity and *in vitro* blood clotting activity of RVV protease isoenzymes in mouse model. The figure (A) shows *in vivo* defibrinogenating activity and figure (B) shows *in vitro* clotting blood of control and protease-treated (2 and 4 mg/kg dose) mice after 6 h i.p. injection. The values are mean ± S.D. of triplicate determinations. Significance of difference with respect to control, *p<0.01; ^†^p<0.001.

All the purified proteases at a dose of 5 mg/kg were found to be non-toxic to mice or house geckos and no adverse effects or behavioral changes were noticed in treated-animals. Light microscopic examination of the lungs, the liver, the kidney and the cardiac tissues from the protease-treated mice did not show any morphological alterations or provide any evidence of intravascular coagulation. Injection of all the four protease isoenzymes induced dose-dependent *in vivo* defibrinogenation of mice plasma (**Fig 6A**) with a corresponding dose-dependent increase in *in vitro* clotting time of blood from treated-mice as compared to blood from control group of mice (**Fig 6B**).

## Discussion

Conversion of prothrombin into its active form thrombin by prothrombinase complex is the central reaction in the blood coagulation process. The rate of prothrombin activation by prothrombinase complex consisting of FXa, Ca^2+^, phospholipids, and factor Va is approximately 300,000-fold higher as compared to prothrombin activation by FXa alone [20]. In this prothrombin activation process, FVa acts as a non-enzymatic cofactor whose function is to enhance the catalytic activity of FXa as well as to promote the binding of both FXa and prothrombin to phospholipids membranes [20]. In the physiological system, thrombin and FXa through limited proteolysis of FV activate it to FVa [16]. However, proteases isolated from many snake venoms such as *D. r. russelii*, *Vipera labetina*, *Naja n. oxiana* have also been characterized to activate circulating factor V which in turn interferes with the normal coagulation process of the victim and/or prey [13],[17],[21].

In the present study, the protease isoenzymes isolated from RVV are found as single chain glycoproteins with slightly different molecular weight. However, the deglycosylated enzymes showed the same molecular mass of ∼23 kDa. This suggests that variations in masses of the native isoenzymes are due to their different degrees of glycosylation which in turn may result in differences in pharmacological potency among these protease isoenzymes. However, the molecular mass of these native glycosylated proteas isoenzymes in the range of 38 to 40 kDa are different than the molecular mass of typical FV activating proteases purified from venom of *D. russelii* (RVV-V, 29 kDa) [10], *V. russelii* (RVV-Vα and RVV-Vγ, 26.1 kDa) [21], *V. lebetina* (VLFVA, 28.4 kDa) [13], *Agkistrodon contortrix contortrix* (26 kDa) [22] and *N. n. oxiana* (48 kDa) [17]. Furthermore, factor V activating proteases viz. RVV-V and VLFVA are reported to contain 6% carbohydrates by mass [10],[13] whereas Contortrixobin is a non-glycoprotein factor V activator from snake venom [22]. In sharp contrast, the four protease isoenzymes in the present study contain a much higher proportion of N-linked carbohydrate (42% to 44% by mass) as compared to other FV activating snake venom proteases. The N-terminal sequences of all the protease isoenzymes (RV-FVP_α_, RV-FVP_β_, RV-FVP_γ_ and RV-FVP_δ_) clearly demonstrate their similarity with the thrombin-like and factor V activating serine proteases from snake venom, and this identification was reconfirmed by the PMF analysis. However, unlike Russelobin which was isolated from the same RVV [7] or other similar thrombin-like serine proteases isolated from other snake venoms, RV-FVP_α_, RV-FVP_β_, RV-FVP_γ_ and RV-FVP_δ_ are devoid of fibrinogen clotting activity or FXa-like activity. Instead they show factor V activating property. Taken together, these observations strongly suggest that these four protease isoenzymes are new: they are previously uncharacterized factor V-activating serine protease isoenzymes from RVV.

The presence of several serine protease isoenzymes is not uncommon in snake venoms. For example, *Bothrops jararaca* venom is shown to contain two isoforms of serine proteases (KN-BJ1 and KN-BJ2) of 38 and 39 kDa, respectively, and their N-terminal sequences are identical [11]. Two basic serine proteases MSP1 and MSP2 purified from the venom of *Bothrops moojeni*, having molecular masses of 34 and 38 kDa, respectively, showed different effects on platelets and TAME [23]. The molecular masses of two pro-coagulant serine proteases, named Cdc SI and Cdc SII purified from venom of *Crotalus durissus cumanensis* were determined as 28,561.4 and 28,799.2 Da, respectively; however, these proteases differ in their clotting activity on human plasma [12]. Similarly, the previously described factor V-activating protease isoenzymes isolated from the venom of *Vipera* (*Daboia*) *r. russelii* also differ slightly in their molecular masses [21]. Although SVSP isoenzymes share a significant sequence similarity, they often show different substrate specificity, and manifest different biological activities and diverse pharmacological profiles [7],[8],[12]. It has been suggested that serine proteases are encoded by multigene family and probably during the process of evolution they emerged by duplication followed by variation of a single ancestral gene [24].

The significant inhibition of protease, BAEE-esterase and coagulant activities of all the four protease isoenzymes under study by classical serine protease inhibitors was achieved by irreversible binding of AEBSF and benzamidine with the serine residue present in the active sites of these enzymes [7],[8]. Furthermore, a parallel decrease in protease, BAEE-esterase and coagulant activities in the presence of serine protease inhibitors as well as monovalent antivenom suggests a correlation among these activities of the protease isoenzymes. The remarkable stability of the four protease isoenzymes against heating and freeze-thawing is in accordance with the previous reports on other serine proteases isolated from snake venoms [7],[13],[25]. It has been shown that the presence of six or more intramolecular disulfide bonds and/or covalently bound carbohydrate moieties is accountable for the high thermostability of SVSPs [7],[13],[25]. Snake venom FV-activating protease isoenzymes or other serine proteases show different substrate specificity and biological activities. For example, unlike RV-FVP_α_, RV-FVP_β_, RV-FVP_γ_ and RV-FVP_δ_, FV activating enzyme VLFVA from *V. lebetina* does not hydrolyze fibrinogen and shows low BAEE hydrolytic activity as compared to the crude venom [13]. The molecular basis of substrate specificity of proteases is quite subtle and involves the contribution of many factors rather than being only dependent on their sequence similarity [7],[8].

On the basis of the chain cleavage pattern, venom fibrin(ogen)olytic enzymes may be classified as either α- or β-chain fibrin(ogen)ases [5]–[8]. Factor V activating RVV proteases in this study preferentially cleaved Aα-chain of fibrinogen with a lower activity towards Bβ-chain of fibrinogen and, therefore, they may be grouped as class A/B serine proteases from snake venom [6]–[7]. Nevertheless, unlike Russelobin [7] and many other thrombin-like serine proteases isolated from snake venom [26], these proteases could not cleave the Arg-Lys bonds on α and β-chains of fibrinogen. Therefore, they failed to liberate fibrinopeptides A and/or B from human fibrinogen. This result reconfirms that *in vitro* procoagulant action of these four protease isoenzymes is not mediated by a thrombin-like mechanism. Furthermore, many of the venom serine proteases are reported to be both fibrinogenolytic and fibrinolytic; however, a number of them are non-fibrinolytic [5],[7]. The RV-FVP_α_, RV-FVP_β_, RV-FVP_γ_ and RV-FVP_δ_ isoenzymes belong to the latter group of proteases as they do not show fibrinolytic activity.

The majority of snake venom procoagulants characterized to date are proteases that specifically activate factor X (FX activators) or prothrombin (factor Xa-like), or convert fibrinogen to fibrin (thrombin-like enzymes [7],[9],[12],[26]. Nevertheless, there is only couple of examples of characterization of factor V activators from snake venom [10],[13],[17]. The absence of amidolytic activity against chromogenic substrate for factor Xa or direct prothrombin activating property of these protease isoenzymes from RVV ruled out their FXa-like mechanism to induce blood coagulation. The RV-FVP_α_, RV-FVP_β_, RV-FVP_γ_ and RV-FVP_δ_ isoenzymes showed higher substrate specificity towards factor V (catalysis takes place within ∼25 to 30 min) as compared to fibrinogenolytic activity (∼120 min) that results in *in vitro* coagulation of blood by these protease isoenzymes. During the initial stage (∼2 min) of prothrombin activation by FXa in presence of RVV-protease/FXa-activated FV and other components of prothrombinase complex, the rate of thrombin generation was directly proportional to FVa concentration [17]. Factor V has very low intrinsic prothrombin-activating ability as compared to FVa; however, with an increase in time, a trace amount of thrombin formed from FXa activated prothrombin leads to significant stimulation of prothrombin activation in a multiplicative way and a correspondingly large increase in the reaction rate [20]. Furthermore, the absence of insulin B-chain degrading ability of these serine proteases is well corroborated with the finding of Reichel et al. [27] showing that venom metalloproteases hydrolyze insulin B-chain much more rapidly than non-thrombin-like serine proteases. Nevertheless, Russelobin, a thrombin-like serine protease from the same venom showed insulin-B chain degradation [7].

Most SVSPs contain varying numbers of glycosylation sites and therefore, the rate of glycosylation differs greatly among these enzymes [7],[8],[25],[26]. The physiological significance or the role of carbohydrate moieties on the activities of SVSPs is still unclear, and many contradictory data have been presented to link the role of carbohydrate moieties in enzyme activity/stability or biological functions. Our study agrees to many other findings in that the deglycosylation of SVSPs resulted in a marked reduction of fibrinogenolytic activity [7],[28] suggesting a significant role played by the carbohydrate moieties of SVSPs in physiological substrate recognition and/or enhancement of catalytic activity of the protease enzymes.

The *in vivo* pharmacological properties of FV activating serine proteases from snake venom have never been explored. The non-toxic nature of RV-FVP_α_, RV-FVP_β_, RV-FVP_γ_ and RV-FVP_δ_ isoenzymes indicates that like Russelobin isolated from RVV [7] and coagulant proteases Cdc SI and Cdc SII purified from *C. d. cumanensis* venom [12], they do not directly induce toxicity in prey. In *in vitro* conditions the four protease isoenzymes demonstrated procoagulant activity owing to their FV-activating property; however, *in vivo* they showed strong anticoagulant effect 6 h after i.p. injection due to dose-dependent defibrinogenating activity and consumption coagulopathy [1],[2],[7].On an average, the venom glands of a large adult RV may store approximately 250 mg venom and therefore, the approximate concentrations of these protease isoenzymes in the blood of mouse and an adult human after a full lethal RV bite will be around 42 to 45 µM and 15 to16 nM, respectively [7]. This reflects the obvious contribution of the RV protease isoenzymes under study in *in vivo* pharmacological effect of RVV in mice or in other rodents. However, pharmacological effects of these protease isoenzymes in RV envenomed victims cannot be ruled out because many relatively weak or non-toxic components of snake venom are reported to interact synergistically to enhance their toxicity or lethality [29].

Hyperfibrinogenemia in blood is associated with increased risk of cardiovascular disorders such as thrombosis and many of the snake venom fibrinogenolytic proteases have been found to induce adverse side effects, rendering them unsuitable for pharmaceutical applications in the treatment of hyperfibrinogenemia [5],[25],[26]. On the contrary, RV-FVP_α_, RV-FVP_β_, RV-FVP_γ_ and RV-FVP_δ_ isoenzymes do not show lethality or toxicity in experimental animals or in *vitro* hemolytic activity, thus supporting their suitability for clinical application as cardiovascular drug [7],[30].

In conclusion, the four protease isoenzymes reported in this study are distinct from previously reported FV activating proteases from RVV or from other snake venoms. The extensive glycosylation provides advantage to the enzymes in showing higher catalytic activity as compared to deglycosylated enzyme. Furthermore, the degree of glycosylation also plays a significant role in physiological substrate recognition and/or enhancement of catalytic activity of the protease isoenzymes. In *in vitro*, they are procoagulant in nature owing to FV activating property and *in vivo* they induce defibrinogenation in mice leading to incoagulable blood.

## Supporting Information

File S1 Supporting tables. Table S1, Summary of purification of protease isoenzymes from venom of *D. r. russelii*. Data represents a typical experiment. Table S2, Peptide mass fingerprinting analysis of four protease isoenzymes purified from venom of *D. r. russelii*.
(DOCX)
