# Supplementary material for: The Pro-Coagulant Fibrinogenolytic Serine Protease Isoenzymes Purified from Daboia russelii russelii Venom Coagulate the Blood through Factor V Activation: Role of Glycosylation on Enzymatic Activity
Source: PLoS One. 2014 Feb 10;9(2):e86823. doi: 10.1371/journal.pone.0086823 (PMC3919717; doi:10.1371/journal.pone.0086823)
Supplement: File S1 — Supporting tables. Table S1, Summary of purification of protease isoenzymes from venom of D. r. russelii. Data represents a typical experiment. Table S2, Peptide mass fingerprinting analysis of four protease isoenzymes purified from venom of D. r. russelii. (DOCX) [file pone.0086823.s001.docx]

**Table S1.** Summary of purification of protease isoenzymes from venom of *D. r. russelii*. Data represents a typical experiment.

| **Fraction** | **Total Protein**  **(mg)** | **Protein Yield**  **(%)** | **BAEE-activity**  **(Units)** | **Specific Activity**  **(Units/mg)** | **Purification**  **(fold)** |
| --- | --- | --- | --- | --- | --- |
| CRVV | 173.3 | 100 | 334815.6 | 1932 | 1.0 |
| GF 58-62 | 9.36 | 5.4 | 46332 | 4950 | 2.6 |
| RV-FVP_α_ | 0.37 | 0.21 | 12256.3 | 33125 | 17.1 |
| RV-FVP_β_ | 0.59 | 0.34 | 87500 | 29750 | 15.4 |
| RV-FVP_γ_ | 0.41 | 0.24 | 6519.8 | 27166 | 14.1 |
| RV-FVP_δ_ | 0.31 | 0.18 | 5316.8 | 29538 | 15.3 |

**Table S2.** Peptide mass fingerprinting analysis of four proteases purified from venom of *D. r. russelii*.

| **Proteases** | **Mr(observed)** | **Mr (Expect)** | **Mr (Cal)** | **Z** | **Peptide** | **Similarity to** | **Snake species** | **% identity** | **Accession No** |
| --- | --- | --- | --- | --- | --- | --- | --- | --- | --- |
| RV-FVP_α_, RV-FVP_β_, RV-FVP_γ_ , RV-FVP_δ_ | 804.6065 | 803.5992 | 804.4606 | 1 | M.FQRLNK.M | Thrombin-like enzyme ancrod (Venombin A) | *Calloselasma rhodostoma* | 66 | [P26324.1](http://www.ncbi.nlm.nih.gov/protein/113827?report=genbank&log$=prottop&blast_rank=1&RID=Y6S75F51211) |
|  |  |  |  |  |  | Thrombin-like enzyme bilineobin | *Agkistrodon bilineatus* | 66 | [Q9PSN3.1](http://www.ncbi.nlm.nih.gov/protein/13959654?report=genbank&log$=prottop&blast_rank=2&RID=Y6S75F51211) |
|  |  |  |  |  |  | Thrombin-like enzyme gyroxin B2.1 | *Crotalus durissus terrificus* | 66 | [Q58G94.1](http://www.ncbi.nlm.nih.gov/protein/82124461?report=genbank&log$=prottop&blast_rank=3&RID=Y6S75F51211) |
|  |  |  |  |  |  | Serine proteinase isoform 6 | *Sistrurus catenatus edwardsi* | 66 | [ABG26972.1](http://www.ncbi.nlm.nih.gov/protein/109254948?report=genbank&log$=prottop&blast_rank=4&RID=Y6S75F51211) |
|  |  |  |  |  |  | Thrombin-like enzyme ancrod-2 | *Calloselasma rhodostoma* | 66 | [P47797.1](http://www.ncbi.nlm.nih.gov/protein/1351938?report=genbank&log$=prottop&blast_rank=5&RID=Y6S75F51211) |
|  | 825.3817 | 824.3744 | 824.4102 | 0 | LLEYCK | thrombin-like enzyme precursor PTLE2 | *Gloydius halys* | 66 | [AAO67554.1](http://www.ncbi.nlm.nih.gov/protein/29293677?report=genbank&log$=prottop&blast_rank=1&RID=Y6T53JG9211) |
|  |  |  |  |  |  | thrombin-like enzyme precursor PTLE3 | *Gloydius halys* | 66 | [AAP15040.1](http://www.ncbi.nlm.nih.gov/protein/30144700?report=genbank&log$=prottop&blast_rank=2&RID=Y6T53JG9211) |
|  |  |  |  |  |  | Snake venom serine protease pallase | *Gloydius halys* | 66 | [O93421.2](http://www.ncbi.nlm.nih.gov/protein/158514815?report=genbank&log$=prottop&blast_rank=3&RID=Y6T53JG9211) |
|  |  |  |  |  |  | Alpha-fibrinogenase shedaoenase/ Snake venom serine protease | *Gloydius shedaoensi* | 66 | [Q6T5L0.2](http://www.ncbi.nlm.nih.gov/protein/158563968?report=genbank&log$=prottop&blast_rank=5&RID=Y6TA0E49211) |
|  | 841.3000 | 840.2927 | 840.3800 | 1 | -.MEFDKR.D + Oxidation (M) | Platelet coagglutinin | *Bothrops jararaca* | 100 | P22030 |
|  |  |  |  |  |  | Botrocetin-2 beta | *Bothrops jararaca* | 100 | [AB794991.1](http://www.ncbi.nlm.nih.gov/nuccore/457866209) |
| RV-FVP_α_ | 1110.8000 | 1109.7927 | 1109.663 | 0 | MPLPVALLTR | Dipeptidylpeptidase 4a | *Gloydius brevicaudus* | 86 | [BAD06332.1](http://www.ncbi.nlm.nih.gov/protein/40363634?report=genbank&log$=prottop&blast_rank=1&RID=Y6UW0PXJ211) |
|  |  |  |  |  |  | venom dipeptidylpeptidase IV | *Demansia vestigiata* | 86 | [ABQ63101.1](http://www.ncbi.nlm.nih.gov/protein/148372355?report=genbank&log$=prottop&blast_rank=3&RID=Y6UW0PXJ211) |
|  |  |  |  |  |  | venom dipeptidylpeptidase IV | *Notechis scutatus* | 86 | [ABQ63105.1](http://www.ncbi.nlm.nih.gov/protein/148372363?report=genbank&log$=prottop&blast_rank=6&RID=Y6UW0PXJ211) |
|  | 1161.900 | 1160.8927 | 1160.9927 | 1 | R.EELAPYPKSK.K | Bradykinin-potentiating and C-type natriuretic peptides | *Protobothrops flavoviridis* | 91 | P0C7P5 |
| RV-FVP_β_ | 6893.0401 | 6892.0328 | 6891.9986 | 0 | K.CDCQGGPNALWSAGATSLDCIPECPYHKPLGFESGEVTPDQITCSNPEQYVGWYSSWTANK.A | Venom coagulation factor Va-like protein | *Oxyuranus microlepidotus* | 68 | Q58L90.1 |
|  |  |  |  |  |  | Venom prothrombin activator pseutarin-C non-catalytic subunit | *Pseudonaja textilis* | 68 | Q7SZN0 |
|  |  |  |  |  |  | Venom coagulation factor Va-like protein | *Oxyuranus scutellatus scutellatus* | 68 | Q58L91 |
| RV-FVP_δ_ | 3561.5188 | 3560.5115 | 3560.7935 | 0 | K.LQLSYMDLYLSHFPVPLQPGEELLLMDAQGK.I + Oxidation (M) | serine protease | *Echis pyramidum leakeyi* | 62 | ADI47546 |
